# A field-based recommender system for crop disease detection using machine learning

**DOI:** 10.3389/frai.2023.1010804

**Published:** 2023-04-26

**Authors:** Jonathan Omara, Estefania Talavera, Daniel Otim, Dan Turcza, Emmanuel Ofumbi, Godliver Owomugisha

**Affiliations:** ^1^Faculty of Engineering, Busitema University, Tororo, Uganda; ^2^Faculty of Electrical Engineering, Data Management & Biometrics, University of Twente, Enschede, Netherlands; ^3^Google, AI for Social Good, Mountain View, CA, United States; ^4^Papoli Community Development Foundation, Tororo, Uganda

**Keywords:** crop disease monitoring, recommendation systems, natural language processing, smart farming, question-answer pairs, food security

## Abstract

This study investigates crop disease monitoring with real-time information feedback to smallholder farmers. Proper crop disease diagnosis tools and information about agricultural practices are key to growth and development in the agricultural sector. The research was piloted in a rural community of smallholder farmers having 100 farmers participating in a system that performs diagnosis on cassava diseases and provides advisory recommendation services with real-time information. Here, we present a field-based recommendation system that provides real-time feedback on crop disease diagnosis. Our recommender system is based on question–answer pairs, and it is built using machine learning and natural language processing techniques. We study and experiment with various algorithms that are considered state-of-the-art in the field. The best performance is achieved with the sentence BERT model (RetBERT), which obtains a BLEU score of 50.8%, which we think is limited by the limited amount of available data. The application tool integrates both online and offline services since farmers come from remote areas where internet is limited. Success in this study will result in a large trial to validate its applicability for use in alleviating the food security problem in sub-Saharan Africa.

## 1. Introduction

Agriculture employs 70% of the population in eastern Africa (FAO, 2010). The farming community in the sub-Saharan region can be divided into two major groups. On one side, most of smallholder farmers (over 70%) grow subsistence crops, such as cassava, sweet potatoes, plantains, beans, and maize. On the other side, there are large-scale farmers who produce mainly cash crops and livestock. The challenge of crop pests and diseases remains a big threat and over 35–40% of yield is lost annually in the sub-Saharan region (Economics, 2018). Current methods of disease detection involve agricultural experts. However, a great shift has been observed from the use of experts to the use of machine learning and computer vision techniques to inspect crops by use of a mobile device (Barbosa et al., 2020; Kakani et al., 2020). Among the rural and smallholder farmers, a mobile phone connects individuals and provides information, market, and services. Therefore, this research is driven on the basis that an extension worker or smallholder farmer can use a mobile phone application for early detection and diagnosis of crop diseases and pest manifestations in the field (Danso-Abbeam et al., 2018; Michael et al., 2019). This study leverages techniques from machine learning to analyze crop images and diagnose crop diseases as well as their severity. This study also builds on initial studies, e.g., the study by Aduwo et al. (2010) and Owomugisha and Mwebaze (2016) that investigated cassava disease from leaf imagery.

The information needs of farmers are always changing and can be seen as an agricultural cycle in different sessions (Mendes et al., 2020). Farmers are always seeking information about their farms or gardens, and usually, they depend on agricultural expert information (Suvedi and Kaplowitz, 2016). In scenarios where this process has been automated, the feedback is not instant (Patel and Patel, 2016). For example, it is explained that it takes approximately 5–7 days before a farmer can get feedback from a diagnostic application (Monitor Uganda, 2019). Lack of real-time information to farmers has contributed to poor farming practices among smallholder farmers, hence high yield losses.

This study proposes a novel approach that relies on natural language processing (NLP) techniques for the support of diagnosis and assessment in real time of in-field crop disease by non-experts. The research aimed at integrating a smartphone-based disease diagnostic application with a real-time feedback tool that farmers can use at any time in their gardens. Computer vision and machine learning (ML) have been used to diagnose crop diseases in crops, e.g., study by Sambasivam and Opiyo (2021) that investigated cassava diseases, crop yield estimation, crop weed identification, and severity estimation among other areas (Kumar et al., 2015; Tripathi and Maktedar, 2020; Mafukidze et al., 2022). Transfer learning and convolutional neural network approach are used on a cassava dataset of 2,756 images comprising three cassava diseases and two types of pest damage (Ramcharan et al., 2017). The studies by Mwebaze and Biehl (2016) and Owomugisha et al. (2021) proposed to rely on prototype-based classification approaches for the detection of cassava diseases from images. Similar approaches exist in the literature, e.g., work by PlantVillage^1^ on crop disease diagnosis. However, the existing mobile diagnostic system with a Q&A component involves an expert in the loop where the questions are answered by farmers or experts in the community. The challenge with such an approach is that different people may have different opinions about a topic, thus applying machine learning will minimize the chances of giving wrong information.

This study makes two primary contributions. First, it demonstrates a mobile field-based recommendation system for crop disease detection. This system is based on a RetBERT sentence embedding approach that works by measuring similarity between text. The developed approach leads to a recommendation system based on the analysis of written text for early warning interventions. Second, it provides a new dataset composed of 3,939 question–answer pairs crowd-sourced from 100 farmers in Uganda. This dataset is publicly available.^2^

The rest of this study is organized as follows. Section 2 gives an overview of the related studies on agricultural systems on Q&A and the use of NLP in real-time feedback systems. Section 3 presents the material and methods that are used in the experiments. Section 4 presents the experiments that were implemented to assess our proposed approach. Results and discussion of the methods are presented in Section 5. Finally, Section 6 presents the conclusion and future work.

## 2. Related works

In this section, related studies in the field of recommendation systems for agriculture and crop disease diagnosis are discussed.

### 2.1. Agricultural systems on question and answer feedback

Real-time feedback systems using natural language are increasingly attracting attention from the machine learning community given the fast development of the field of human–robot interaction. AgriBot (Jain et al., 2019) presents an Agriculture-Specific Question Answer System that answers questions related to weather, market rates, plant protection and government schemes in India. The system is based on sentence embedding and entity extraction. Several studies have been introduced with the aim of helping farmers in rural areas in India. For example, FarmChat was introduced by Jain et al. (2018). The conversational agent system answers farmers' information needs by picking a question in form of audio and converting it to text in order to provide the most appropriate answer to the farmer. Similarly, TalkBot (Vijayalakshmi and Pandimeena, 2019) uses a speech synthesis web API to provide voice-based responses to farmers in India. A region-specialized system for India was proposed by Yashaswini et al. (2019). The Smart Chatbot agriculture tool uses a K-nearest neighbor algorithm to analyze new patterns in markets, rainfall, seasons, and soil types. E-AGRO (Ekanayake and Saputhanthri, 2020) uses Artificial Intelligence Markup Language (AIML) implemented in a cloud platform to provide responses to farmers. Even though the obtained results by the above-mentioned methods are promising, the existing applications cannot work for some local communities due to the fact that the datasets used to train the models were extracted from specific countries, that is to say, India and farmers need different types of information based on the type of crops grown in their regions.

### 2.2. Works on NLP and real-time feedback

Conversational AI systems are commonly known as chat-bots (Lokman and Ameedeen, 2019; Peng and Ma, 2019). They are intelligent models that can be further categorized into either retrieval-based (Wu et al., 2016; Yan et al., 2016; Tao et al., 2019) or generative models (Sheikh et al., 2019; Kapočiūtė-Dzikienė, 2020; Kim et al., 2020), or even as a combination of both (Yang et al., 2019). The retrieval-based models pick a response from a collection of responses based on the query. The generative models work by generating a response, word-by-word based on the query given, hence the models are prone to grammatical errors. One of the known challenges of generative models is that they are hard to train since they require to learn the proper sentence structure. However, once trained, the generative models tend to outperform the retrieval-based models in terms of handling previously unseen queries and creating an impression of talking with a human (Sojasingarayar, 2020).

In the study presented by Su et al. (2017), the authors introduced an LSTM-based multi-layer embedding for elderly care. The system involved collecting chitchat dataset from daily conversations with the elderly, converting it into patterns, then an LSTM-based multi-layer embedding model was used to extract the semantic information between words and sentences in a single turn with multiple sentences when chatting with the elderly. Finally, the Euclidean distance was employed to select a proper question pattern, which is further used to select the corresponding answer to respond to the elderly.

The study by Singh et al. (2018) presented a chat-bot for small businesses. The system was built on TensorFlow and included machine learning at its core. The process uses TensorFlow to make a neural network and train it with intent file to generate a response model. The response model is, then, used to predict the response from the query of the user. This system consists of three main parts as follows: 1) user interface, 2) neural network model and NLP unit, and 3) feedback system. The study by Mathew et al. (2019) proposed a chat-bot for disease prediction and treatment recommendation. The engine consists of the well-known machine learning algorithm K-nearest neighbor algorithm (KNN). This study indicated that a medical chat-bot can diagnose patients through the analysis of simple symptoms.

The chat-bot for e-learning introduced by Colace et al. (2018) presents the realization of a chat-bot prototype for supporting students during their learning activities. The study introduced two frameworks as follows: 1) the automatic identification of the students' needs due to the adoption of Natural Language Processing Techniques and 2) the selection of the best answer due to the use of the ontological representation of the knowledge domain. Qiu et al. (2017) proposed an AliMe Chat as a sequence to-sequence and Re-rank-based chat-bot engine. The hybrid system combines both generative and retrieval models to provide feedback and uses an attentive Seq2Seq model to optimize the joint results of information retrieval(IR) and generation models. The described techniques show that most of the existing systems either suffer from a generative model problem or a retrieval model problem.

## 3. Materials and methods

### 3.1. The question and answer dataset

Due to the non-existence of a database containing agronomic questions and answers on staple food crops such as cassava, maize, and beans in sub-Saharan Africa, the current approach contributed to the collection of data from farmers. The mobile-based diagnostic application with real-time information was tested in a community with 100 farmers participating in the pilot. Farmers participated by performing diagnostics on cassava (Manihot esculenta Cranz) affected by four major conditions as follows: cassava brown streak disease (CBSD), cassava mosaic disease (CMD), cassava bacterial blight (CBB), and cassava green mite (CGM), as well as healthy plants. Through the diagnostic tool, they also asked questions that surround three main crops (cassava, maize, and beans). Furthermore, the study carried out interviews with the farmers who had no smartphones to acquire more information and the challenges they faced with their crops. The interviews were conducted over 5 days. Each day, 10 farmers were interviewed, and a minimum of 10 questions were generated from each farmer. The areas surveyed included villages in the Kole district in northern Uganda and by the end of the exercise, a total of over 500 questions had been obtained. Of the 50 farmers who contributed to the Kole district, 26 farmers were male while 24 were female, and their ages ranged between 22 and 70 years. However, one of the biggest challenges was that some of the farmers were not comfortable sharing their information, and it was difficult to elicit questions from them. Therefore, the study implemented the first version of the application with the proposed method in Section 3.2, gave it to farmers to interact with it, as much as possible and their questions were stored in a database. Thus, the approach of farmers sending questions *via* their smartphones became flexible and convenient to both farmers and the receiving team. The data collected were then assigned to the agricultural experts for annotation and answers. The data collected from farmers through the application and the one collected from farmers through interviews were concatenated as a single dataset. Table 1 shows a sample of questions and answers extracted from this dataset.

**Table 1:** A sample of 10 questions (Q) and their corresponding answers (A) from experts are extracted from the training dataset.

**No**.	**(Q)uestion and (A)nswer**
1	Q. What should I do to the beans if the weather suddenly turns to hot or dry after planting
	A. Carry out mulching
2	Q. How can I prevent pest and diseases in maize
	A. By spraying with pesticides and practicing crop-rotation also helps to reduce on pests and diseases
3	Q. When should I spray maize after planting
	A. After 1 month
4	Q. Why is maize not doing well in a rocky place
	A. Rocky places have low nutrient content and low water content
5	Q. Why are there always termites in the hole which I had dug for planting cassava
	A. This can be because it's soft and wet
6	Q. Why is the stem delaying to germinate?
	A. Maybe it was young or covered with a lot of soil
7	Q. Why is the planted cassava stem drying up before germinating?
	A. It could've been young or it was destroyed by termites
8	Q. Why is there no cassava in the roots?
	A. This is a result of cassava brown streak virus
9	Q. How can I make sure that the bean seeds germinate?
	A. By braking seed dormancy prior to planting
10	Q. Why are some bean plants drying up even in the rainy season?
	A. This is caused by root rots

**Figure 1:**
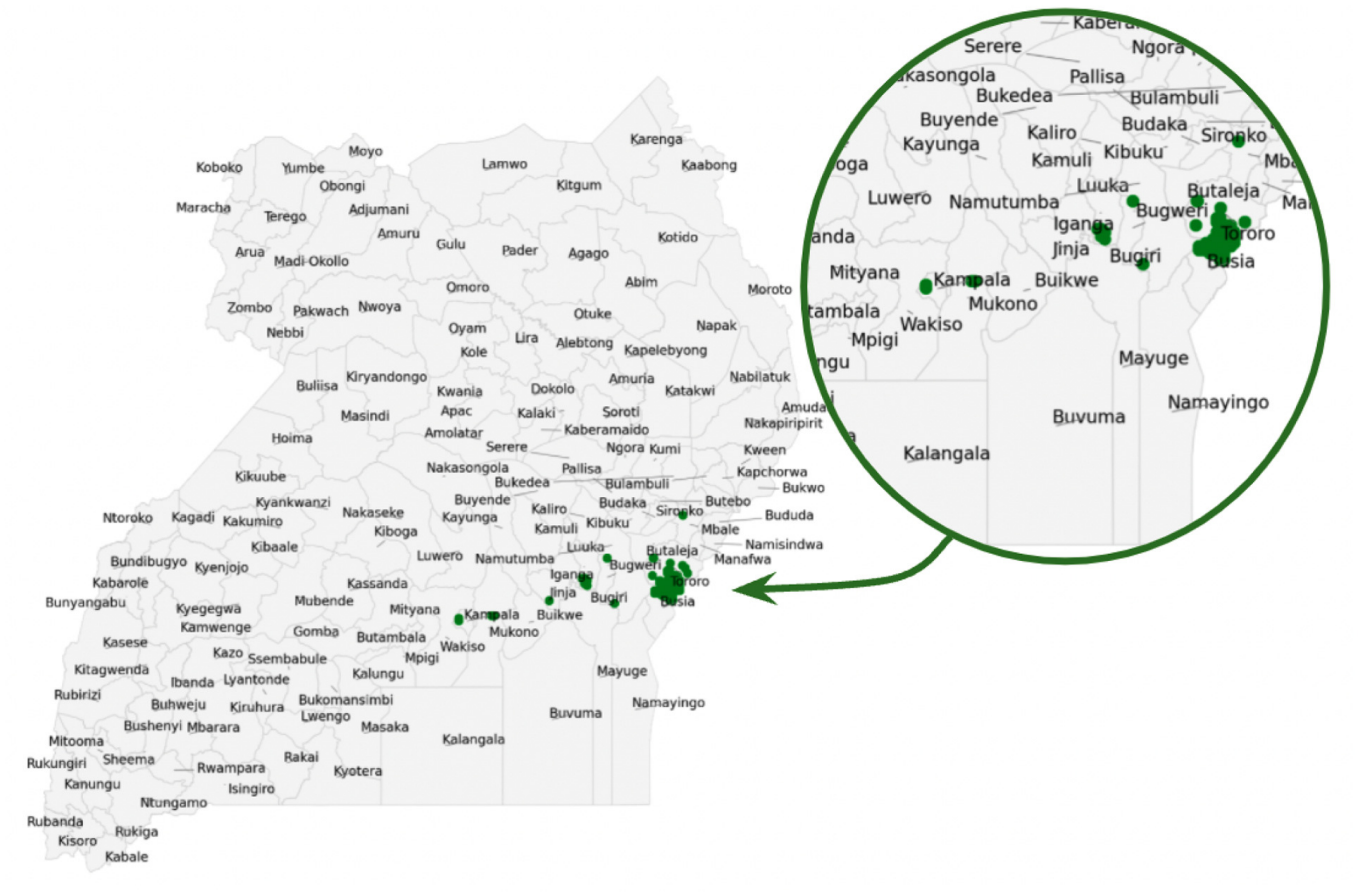
Data collection in eastern Uganda. Zoom-in highlights the districts represented by green spots. Green spots indicate the regions that participated in the data collection.

Figure 1 presents data coming from districts of Tororo and Busia (the most participating regions) and neighboring districts: Sironko, Butaleja Bugweri, Bugiri, and Iganga in the eastern region of Uganda. Other data coming in from the city centers: Kampala and Mukono. The green spots represent the areas of data collection within the district. The distribution of questions for the three crops with the majority of the questions asked on cassava crop (56%) while maize (16%) and beans (28%) had the least representation.

#### 3.1.1. Data pre-processing

The data pre-processing stage was carried out to prepare the dataset for model training. The following steps were involved.

1. *Spelling correction, white space, and punctuation removal:* This involves reading through the sentences and correcting the miss-spelled English words. Grammatical errors both on the questions collected and the answers provided by the experts were checked as well as ensuring answers addressed the question appropriately. Punctuation and white space removal processes were automated.
2. *Rephrasing answers and changing to lowercase:* The process aimed at shortening the answers, also rephrasing the answers by checking grammatical errors. Spelling correction was performed manually by reading through each sentence. In addition, all letters were lowercased, and this task was automated.
3. *Removing unanswered questions:* Some questions could not be answered by the experts since they were not clear and understood due to misspelled words. To have a clean dataset for training model, the rows with missing answers were dropped.

### 3.2. The RetBERT framework training

Here, we briefly describe the machine learning framework employed, Sentence-BERT (SBERT) (Reimers and Gurevych, 2019). SBERT is a modification of the pre-trained BERT network (Devlin et al., 2018). To fine-tune the BERT, siamese and triplet networks were created to update the weights such that the produced sentence embeddings are semantically meaningful and can be compared with cosine similarity.

*Model Architecture*: The SBERT technique outputs sentence embeddings that are semantically meaningful and are later used to perform similarity search and ranking. SBERT works by adding a pooling operation to the output of BERT to derive a fixed-sized sentence embedding. A three pooling strategies are described in the literature. In this study, we used a default configuration (MEAN strategy) which computes the mean of all output vectors.

*Objective function*: The original SBERT network structure by Reimers and Gurevych (2019) proposes three objective functions, such as classification, regression, and triplet objective function. As mentioned, the proposed SBERT architecture uses a fine-tuned BERT in a siamese/triplet network. Given an anchor sentence *a*, a positive sentence *b*, and a negative sentence *c*, triplet loss tunes the network such that the distance between *a* and *b* is smaller than the distance between *a* and *c*. This loss function is mathematically described in Equation (1) as follows:


(1) $$\begin{array}{l} \max\left(||s_{a}-s_{b}||-||s_{a}-s_{c}||+\epsilon ,0\right) \end{array}$$

where *s*_*x*_ the sentence embedding for *a*/*c*/*b*, ||·|| a distance metric, and margin ϵ. Margin ϵ ensures that *s*_*b*_ is at least ϵ closer to *s*_*a*_ than *s*_*c*_, where the Euclidean distance is set to ϵ = 1 in the default setting.

**Figure 2:**
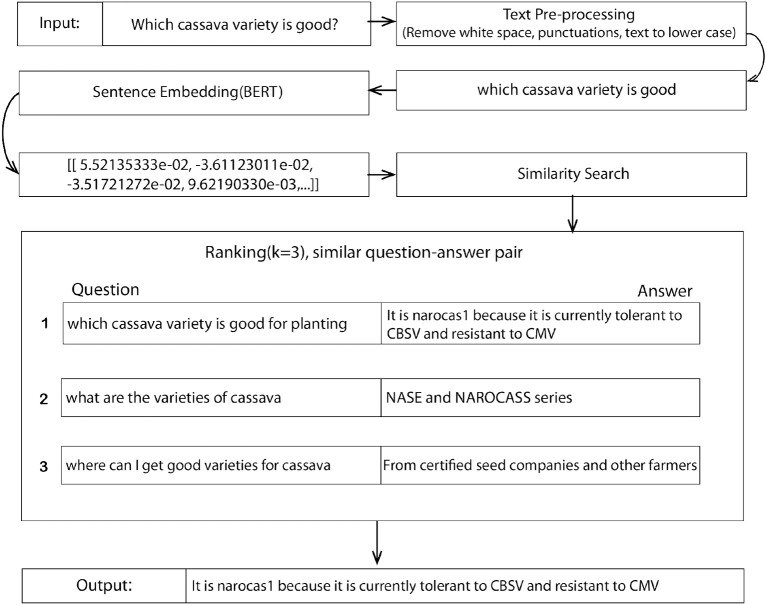
The proposed retrieval-based architecture (RetBERT) with a sentence embedding and ranking given an example input and the resulting output.

*Training details*: The data obtained were pre-processed following steps presented in Section 3.1.1, and subsequently trained on three techniques, such as RetBERT, Seq2seq, and Haystack. The proposed model techniques were chosen for their simplicity in solving semantic search problems than hungry top-performing models that require huge datasets. The following steps describe the proposed RetBERT model architecture from the data capture to the final output. This process is also presented in Figure 2.

1. *Question as input:* A question is taken in the form of text with no more than 256 characters. It, then, goes through a text pre-processor stage by converting it to lowercase, removing the punctuation as well as whitespace in the sentence if present. The text pre-processing stage showed a 5% increase in the model performance.
2. *Sentence Embedding (SBERT):* The SBERT model described above is then applied. The model encodes the pre-processed question into a dimensional vector of shape (384) and passes it to the similarity search function.
3. *Similarity search and Ranking:* A cosine similarity was applied to measure the similarity between vectors. This process returns a sorted array of values ranked by how similar the input question is to the existing questions in the training dataset. The model uses the *k* values to rank the question-answer pairs at index *n* as 1, 2, and 3 as the top three answers. Finally, the answer to the question at rank 1 is returned as the answer to the input question. For training and testing, a total of 3,939 question–answer pairs were used. Of this data, 90% of the data were used for training and 10% for testing. The batch size was 64 and the epoch size was 100.
4. *Deployment—Integration into a mobile App:* The retrieval model with a sentence BERT model was converted into an API and deployed into Google Cloud App Engine using Python (Flask). In addition, an Android app was built to access the model API. With this approach, farmers were required to have an internet connection. However, since farmers live in remote areas where internet connectivity is limited, an offline version of the Q&A system was deployed and built from PyTorch Mobile and TorchScript. The answers are still similar semantically, and typically, the selected answer from one model is in the top three responses in the other model. Given the highly similar performance of the two models, the tool was set to use only the offline model as default rather than switching between the two based on internet connectivity, which is error-prone and could lead to a frustrating and inconsistent user experience. Thus, the models will be periodically updated when there is a large influx of new questions from farmers and answers from experts.

### 3.3. Crop disease diagnosis

**Figure 3:**
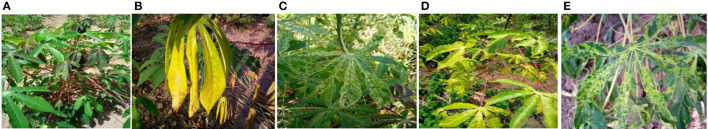
Sample images associated with the cassava disease classes that trained the diagnostic component. **(A)** Healthy, **(B)** CBB, **(C)** CGM, **(D)** CBSD, and **(E)** CMD.

**Figure 4:**
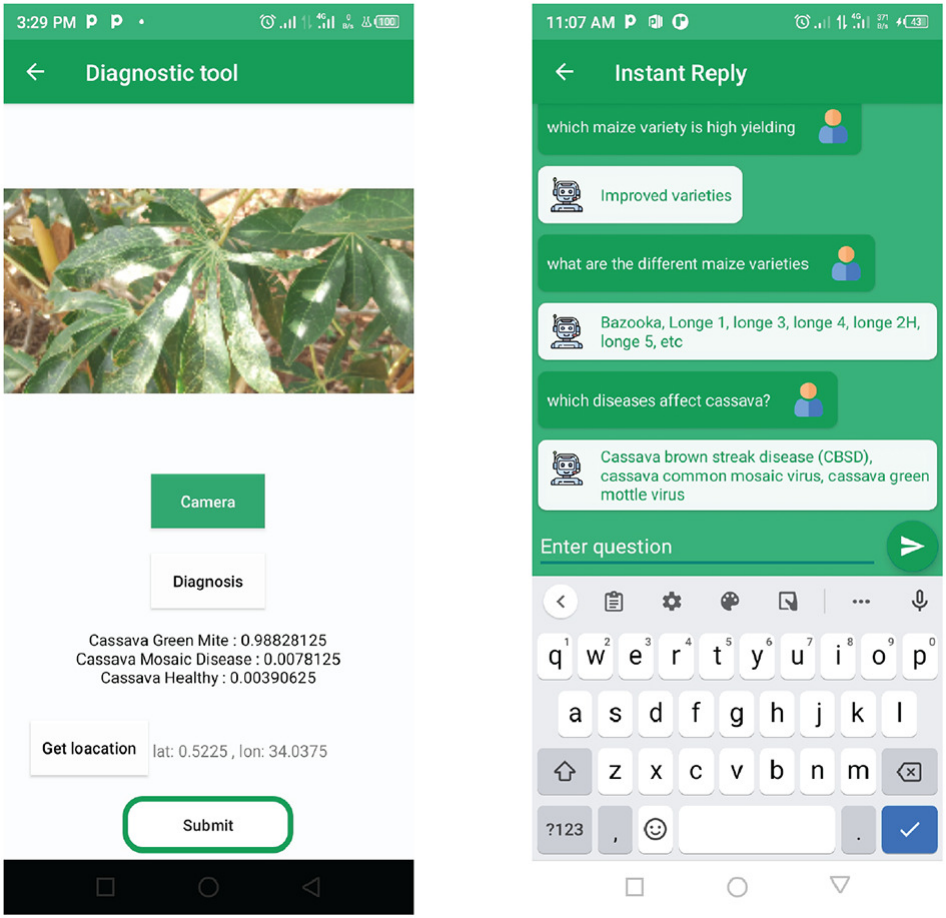
The user interfaces that a farmer uses to diagnose cassava crop disease and ask agronomic questions.

The task of crop disease diagnosis has been previously addressed. The authors in Mwebaze et al. (2019) presented the iCassava 2019 Fine-Grained Visual Categorization Challenge. Thus, the disease diagnosis component of this study builds on iCassava 2019 dataset^3^ and follows the implementation at: https://tfhub.dev/google/cropnet/classifier/cassava_disease_V1/2 (accessed on 31 December 2022) with less modification. The open-source code model uses TensorFlow Lite, an efficient model format that can easily be deployed in embedded systems with limited hardware resources. The model is trained on 9,430 cassava leaf images under five categories, such as Healthy, Cassava Mosaic Disease (CMD), Cassava Bacterial Blight (CBB), Cassava Greem Mite (CGM), and Cassava Brown Streak Disease (CBSD). Figure 3 shows examples of cassava leaf images from the dataset for the above classes. The model uses MobileNetV3 architecture (Yang et al., 2018; Howard et al., 2019) and attains a classification accuracy of 88% for cassava disease detection. The model was deployed as a mobile application on an android smartphone. This uses android ML Kit^4^ that exposes the model as an on-device API. When a leaf image is taken by the phone camera, it is processed and passed to the model predict function as a bitmap image. The model processes the image and returns the predicted probabilities and their class names, as shown in Figure 4.

## 4. Experiments

This section describes the experimental setup that was used to assess the performance of the proposed recommendation framework.

### 4.1. Evaluation metrics

This study applied a BLEU score metric by Papineni et al. (2002) to evaluate the performance of the models. The metric has been applied in related studies in translation models, e.g., Vijayalakshmi and Pandimeena (2019), Yashaswini et al. (2019), Qiu et al. (2017), and Yan (2018). BLEU score metric is given by the formula as follows:


(2) $$\begin{array}{l} \begin{array}{c} BLEUscore=\frac{\sum M_{max}}{W_{t}} \end{array} \end{array}$$

where *M*_*max*_ represents the maximum clip count of the number of times a word (uni-gram) in the model answer appears in the reference answer (expert answer), and *W*_*t*_ is the count of words (uni-grams) in the model answer. For example, given the question “*What caterpillars are affecting maize in the garden?,“* the expert answer is as follows: “*They are fall army worms and stem borers.“* If the model gives the following answer, “*Fall army worms or larvae,“* the BLUE score can be computed as follows:


$$\begin{array}{l} BLEUscore=\frac{\sum M_{max}}{W_{t}}=\frac{1+1+1+0+0}{5}=0.6 \end{array}$$

The BLEU score is a value that ranges between 0 and 1, measuring the similarity of a model answer to an expert answer. The higher the BLEU score, the better the model. In python programming, the BLEU score can be accessed through the NLTK package by importing *sentence*_*bleu*, and the BLEU score can be calculated as follows:


$$\begin{array}{l} sentence\text{\_}bleu\left(x,y,weights=\left(1,0,0,0\right)\right), \end{array}$$

where *x* is the model answer, *y* is the expert (reference) answer, and *weights* = (1, 0, 0, 0) for a uni-gram which compares word by word in each of the two sentences. The BLEU score is later treated as a percentage. Moreover, we also evaluate the computational cost for a system that needs to be deployed on a mobile device.

### 4.2. Baseline methods

To assess the performance of the proposed approach, two state-of-the-art models for conversational AI were implemented as follows:

- Seq2seq model (Qiu et al., 2017). This is implemented using an encoder with a Bi-directional Long Short Term Memory (LSTM) layer with 256 cells, an attention layer, dropout (rate = 0.3), and a Long Short Term Memory decoder layer with 512 cells. Both the encoder layer and decoder layer have an embedding layer connected to them with an output dimension of 1,024, a return sequence set to true given that the states of the cells are required. These layers are connected as follows: the decoder layer is connected to the attention layer, which is followed by a dropout layer. Finally, an output dense layer is added to it.
- Haystack (Landsberg and Michałek, 2019). Here, the study implemented a FAISSDocumentStore document store with parameters vector_dim = 128 and faiss_index_factory_str = “Flat” to output text vector embeddings of dimension = 128. The file conversion preprocessor was set as PreProcessor(clean_empty_lines = True, clean_whitespace = True, clean_header_footer = True, split_by = “word”, split_length = 200, split_respect_sentence_boundary = True) and used to add the processed file data as a list of json text data to the document store. All these were put in a pipeline with a sequence generator that was responsible for generating full sentences as feedback. In addition, Haystack was initially used to retrieve information from text documents^5^ that hold information on cassava, maize, and beans. This dataset is part of the pipeline for Section 3.1.1.

### 4.3. Implementation details

*Training details*: The proposed RetBERT Section 3.2 and baseline models Section 4.2 were trained on 3,939 question–answer pairs. The machine learning procedure started with data shuffling, a step that was performed prior to model training in order to create more representative training and testing sets. The model uses 90% and 10% for training and test sets, respectively.

This study was implemented using Python 3.7 with an Intel(R) Xeon(R) CPU @ 2.20GHz and RAM of 12.68 GB. We used TensorFlow version 2.8.2.

## 5. Results and discussion

This section presents results obtained from a recommendation system based on the RetBERT model and the other two baseline models on the same dataset.

The results in Table 2 present the quantitative performance of these three models in terms of the average BLEU score accuracy achieved. The results show that RetBERT had the highest average performance of 50.8%, Seq2seq had 46.9% while HayStack had the lowest of 31.9%. The standard deviations of the responses of the models were 29.6%, 19.9%, and 28.8% for RetBERT, HayStack, and Seq2Seq, respectively (Table 2). The presented the RetBERT model obtained an accuracy that represents an improvement of 3.9% and 18.9% with respect to the Seq2Seq and HayStack models, respectively. The RetBERT model performs better when retrieving answers from an existing dataset as compared with the baseline methods. Thus, standard deviations of 29.6%, 19.9%, and 28.8% applied to RetBERT, HayStack, and Seq2seq models, respectively. This results in low performance when it comes to predicting answers to questions that have no similar entries in the training dataset. For the Seq2Seq model to perform well, it should have seen something similar to the question being asked. HayStack also has the lowest average performance; however, the model scored more similarly across all questions as shown by a lower standard deviation of 19.9%.

**Table 2:** Quantitative performance of the evaluated retrieval models RetBERT, HayStack, and Seq2seq.

**RetBERT**	**HayStack**	**Seq2seq**
**BLUE Score**		
50.76 ± 29.57	31.87 ± 19.90	46.85 ± 28.84
**Computational cost (ms)**		
0.06 ± 0.02	13.87 ± 5.99	0.67 ± 0.42

We report the average and standard deviation obtained when applied to our collected dataset.

To assess the performance of the models on hardware resources, the time taken during inference was calculated in milliseconds for each of these three models. This was performed using the test dataset, and the results are presented as computational costs in Table 2. For the HayStack model, more time was taken during inference, and more outliers were observed as compared the RetBERT and Seq2seq models. The result is obtained from querying a large text document during inference. The RetBERT model has a lower computational cost and can easily be deployed to run even on low-resource hardware devices. HayStack is the most computationally expensive model; thus, it would require more time and memory. Finally, the models were deployed on a mobile device with a user-friendly interface (Figure 4) to enable users to interact with these models on a mobile phone. A sample of five Q&A pairs from the test dataset was used to compare the model outputs with the original answers. These results are presented in Table 3, with a column containing the questions (Q) and original answers (A) and a column containing the answers returned by each of these models during inference. A total of two things were observed as follows: 1. Some answers had short sentences and others had lengthy sentences; and 2. Some answers were correct and others were not. In this set of samples, the RetBERT model got three out of five answers correct that were very similar to the original answers, and the HayStack model had the worst performance, getting only two out of five answers correct that were close to the original answers. However, its answers were also lengthy, which could have resulted from the fact that the HayStack (Landsberg and Michałek, 2019) model is querying a large text document for answers and has a sequence generator that does not perform well when it comes to generating short sentences.

**Table 3:** **Left column:** Sets of sample questions (Q) and their original answers (A) extracted from the test dataset.

**(Q)uestions and original (A)nswers in the test dataset**	**Predicted answers by RetBERT, HayStack and Seq2Seq (S2S)**
Q. Which criteria should i use to keep my cassava healthy? A. Use clean seeds	**RetBERT:** Good soil and weather conditions **HayStack:** Keep the cuttings out of the sun and in an air-tight container. Don't leave them exposed to the elements. **S2S:** About 10 cm
Q. How many maize seeds should i plant per hole? A. One or two	**RetBERT:** One to 2 seeds **HayStack:** The recommended planting depth is 2.8: > The speed at which the seed germinates and emerge out of soil depends on the soil fertility, plant type and growth habits, rainfall, and temperature. **S2S:** One to 2 seeds
Q. What is the major economic benefit of cassava growing to the government? A. Food security	**RetBERT:** Food and income security **HayStack:** It's not a major economic benefit. It's a major political benefit. **S2S:** Cassava that is high yielding and disease resistant and tolerant
Q. When should i start spraying my cassava? A. When pests appear	**RetBERT:** Depending on what you are spraying if weeds, when plants are mature enough **HayStack:** It depends on the type of cassava you're talking about. If it's the kind that grows on the side of the road, you should spray it before you go anywhere near it. Otherwise, it's best to leave it alone. **S2S:** Depending on what you are spraying if weeds when plants are mature enough
Q. What fertilizer can be used in beans? A. Npk, urea	**RetBERT:** Npk, urea **HayStack:** Fertilizer can be used in beans as long as it has the right amount of nitrogen, phosphorus, and potassium in it. **S2S:** Nitrogen fertilizers are commonly used

Right column: Samples of the predicted answers by the different models given a question (Q) in the left column during inference.

Overall, the performance of recommender-based applications is dependent on the size of the dataset. Initially, the recommender tool was built on 500 question–answer pairs with BLEU score accuracy below 40%. The performance gradually improved, as more questions were crowd-sourced from farmers over the period of 10 months obtaining 3,939 question–answer pairs at 50.8% accuracy. Although the performance accuracy of the system is not so high, this research creates a path for a recommendation system in agriculture where data are not available, and expert knowledge has been limited.

## 6. Conclusion

This research presents a recommendation system based on the analysis of written text information. The system was used by smallholder farmers in a rural community in eastern Uganda. The ultimate goal of this study was to equip farmers with a diagnostic tool on their smartphones that they can diagnose without waiting for expert visits. The flexibility of the tool allows farmers to move with their phones anywhere and can still access the application. The deployed application had both online and offline (on-device) options considering that most farmers live in remote areas where internet connectivity is limited, with similar performance between the two models.

The study contribution is two-fold; a new dataset that describes the interaction between farmers and an intelligent system presented, and a recommendation system based on text retrieval. Several methods are compared, including Seq2Seq, HayStack, and RetBERT. The BLEU score is used to assess performance, indicating the robustness of RetBERT, which achieves a score of 50.8%. The study concluded that the RetBERT model was sufficient for the field-based trial model. The key motivation for our approach is that it can work in low-resource environments or on low-computational power systems, but most importantly, it can perform recommendations, thus substituting the physical presence of experts. More study in this field will demand a higher amount of data given the current data hungry top-performing model.

Our future study will go in the following directions; (i) Validating our diagnostic model with another group of farmers and experts. This process will increase the diversity in terms of crop data and farmers, information, and it will, in turn, improve our question-and-answer model. (ii) Exploring the source of the differences between the models, e.g., converting the model to fundamental differences in precision in Python vs. Java computation. (iii) Combining different datasets in the Q/A model, e.g., text, visuals, and audio. The approach is motivated by the fact that most farmers use mobile phones that can collect images, videos, and audio, which we hypothesize can support the final recommendation.

In conclusion, the smartphone mobile diagnostic tool with real-time feedback comes with various benefits as follows: (i) Farmers do not have to wait for experts, as they can get instant advice on their gardens on three major crops, such as cassava, maize, and beans. (ii) The findings from this study paves way for the agricultural recommender systems in developing worlds by improving the livelihoods of smallholder farmers through early intervention measures, thus alleviating the food security problem in sub-Saharan Africa.

## Data availability statement

The original contributions presented in the study are publicly available. This data can be found here: https://github.com/JonaOmara/AgroQA-Dataset/blob/main/AgroQA%20Dataset.csv.

## Ethics statement

Written informed consent from the farmers was required to participate in this study in accordance with the national legislation and the institutional requirements.

## Author contributions

JO and DT: analysis and interpretation of results. ET, DO, and GO: study conception, design, and manuscript preparation. EO: data collection. All authors reviewed the results and approved the final version of the manuscript.

## Footnotes

^1^ https://play.google.com/store/apps/details?id=plantvillage.nuru&hl=en

^2^ https://github.com/JonaOmara/AgroQA-Dataset/blob/main/AgroQA%20Dataset.csv

^3^ https://www.kaggle.com/competitions/cassava-disease/data

^4^ https://developers.google.com/ml-kit

^5^ https://www.agriculture.go.ug/crop-sub-sector-publications/
